# Supplementary material for: Zygotic gene activation in the chicken occurs in two waves, the first involving only maternally derived genes
Source: eLife. 2018 Oct 30;7:e39381. doi: 10.7554/eLife.39381 (PMC6242549; doi:10.7554/eLife.39381)
Supplement: Transparent reporting form [file elife-39381-transrepform.pdf]

eLIFE
1st Floor
24 Hills Road
Cambridge CB2 1JP, UK
P 01223 855340
W elifesciences.org
T @elife

eLife's transparent reporting form

We encourage authors to provide detailed information *within their submission* to facilitate the interpretation and replication of experiments. Authors can upload supporting documentation to indicate the use of appropriate reporting guidelines for health-related research (see EQUATOR Network), life science research (see the BioSharing Information Resource), or the ARRIVE guidelines for reporting work involving animal research. Where applicable, authors should refer to any relevant reporting standards documents in this form.

If you have any questions, please consult our Journal Policies and/or contact us: editorial@elifesciences.org.

Sample-size estimation

- You should state whether an appropriate sample size was computed when the study was being designed
- You should state the statistical method of sample size computation and any required assumptions
- If no explicit power analysis was used, you should describe how you decided what sample (replicate) size (number) to use

Please outline where this information can be found within the submission (e.g., sections or figure legends), or explain why this information doesn't apply to your submission:

The sample size was determined by 3 replications per experimental condition to be compared by reference to similar studies published in other organisms (Heyn et al., 2014, Cell Rep, Anderson et al., 2017, Dev Cell, Chen et al., 2017, Plant Cell). In this study, 6 whole genome sequencing data (3 female White Leghorn and 3 male Korean Oge), 9 single embryonic RNA sequencing data (triplicates at oocyte, zygote, and EGK.X stage), and 21 bulk embyonic RNA-seq data (triplicates at oocyte, zygote, EGK.I, EGK.III, EGK.VI, EGK.VIII, and EGK.X) were used. As this study included the first attempt at a chicken early embryo, sample size calculation was not performed through power analysis. More details can be found in Materials and methods.

Replicates

- You should report how often each experiment was performed
- You should include a definition of biological versus technical replication
- The data obtained should be provided and sufficient information should be provided to indicate the number of independent biological and/or technical replicates
- If you encountered any outliers, you should describe how these were handled
- Criteria for exclusion/inclusion of data should be clearly stated
- High-throughput sequence data should be uploaded before submission, with a private link for reviewers provided (these are available from both GEO and ArrayExpress)

Please outline where this information can be found within the submission (e.g., sections or figure legends), or explain why this information doesn't apply to your submission:

Whole genome sequencing data was generated from (3 male Korean Oge and 3 female White Leghorn) and three randomly mated pairs are biological replications. In case of whole RNA-sequencing data, biological triplicates were assigned at each developmental stage. Whole genome sequencing data was deposited in SRA and RNA-seq data was uploaded in GEO database.

Statistical reporting

- Statistical analysis methods should be described and justified
- Raw data should be presented in figures whenever informative to do so (typically when N per group is less than 10)
- For each experiment, you should identify the statistical tests used, exact values of N, definitions of center, methods of multiple test correction, and dispersion and precision measures (e.g., mean, median, SD, SEM, confidence intervals; and, for the major substantive results, a measure of effect size (e.g., Pearson's r, Cohen's d)
- Report exact p-values wherever possible alongside the summary statistics and 95% confidence intervals. These should be reported for all key questions and not only when the p-value is less than 0.05.

Please outline where this information can be found within the submission (e.g., sections or figure legends), or explain why this information doesn't apply to your submission:

Negative-binomial based RNA-seq analysis was performed to consider over-dispersion issue. We used 5% significance level in hypothesis test after multiple testing adjustment using Benjamini and Hochberg's method. Further information of statistical model can be found in Materials and methods.

(For large datasets, or papers with a very large number of statistical tests, you may upload a single table file with tests, Ns, etc., with reference to sections in the manuscript.)

Group allocation

- Indicate how samples were allocated into experimental groups (in the case of clinical studies, please specify allocation to treatment method); if randomization was used, please also state if restricted randomization was applied
- Indicate if masking was used during group allocation, data collection and/or data analysis

Please outline where this information can be found within the submission (e.g., sections or figure legends), or explain why this information doesn't apply to your submission:

Three female White Leghorn (WL) and three male Korean Oge (KO) were randomly mated. The egg-laying times of female WL were recorded. A single hybrid EGK.X blastoderm was collected from WL hens after oviposition. To collect single oocytes and hybrid zygotes, WL hens were sacrificed and the follicles were collected. Oocytes and hybrid zygotes were collected simultaneously from one WL hen. Only zygote embryos not showing cleavage and located in the magnum were collected within 1.5 h after fertilisation according to the recorded egg-laying times. As this study is not randomized trial, blind design was not used.

Additional data files ("source data")

- We encourage you to upload relevant additional data files, such as numerical data that are represented as a graph in a figure, or as a summary table
- Where provided, these should be in the most useful format, and they can be uploaded as "Source data" files linked to a main figure or table
- Include model definition files including the full list of parameters used
- Include code used for data analysis (e.g., R, MatLab)
- Avoid stating that data files are "available upon request"

Please indicate the figures or tables for which source data files have been provided:

We attached the python code used for RNA-seq quantification analysis in Supplementary file 6.
